# Endoscopic ultrasound–guided ethanol ablation for aldosterone-producing adenoma

**DOI:** 10.1055/a-2875-0099

**Published:** 2026-06-11

**Authors:** Liang Deng, Yonggang Hao, Xiangshuang Zhang, Qifu Li, Jinbo Hu, Shumin Yang

**Affiliations:** 1 Department of GastroenterologyThe First Affiliated Hospital of Chongqing Medical UniversityChongqingChina; 2 Department of AnesthesiologyThe First Affiliated Hospital of Chongqing Medical UniversityChongqingChina; 3 Department of Endocrinology 117972 The First Affiliated Hospital of Chongqing Medical UniversityChongqingChina

A 39-year-old woman with a 4-year history of hypertension presented with recurrent hypokalemia for 1 month. Biochemical testing confirmed the diagnosis of primary aldosteronism. Unenhanced computed tomography (CT) showed a 22×13 mm low-density nodule in the left adrenal gland, and ^68^ Ga-Pentixafor positron emission tomography (PET)/CT demonstrated focal increased tracer uptake within the lesion, consistent with a functional adenoma ( **Fig.** 1 ). ​1​

**Fig. 1:**
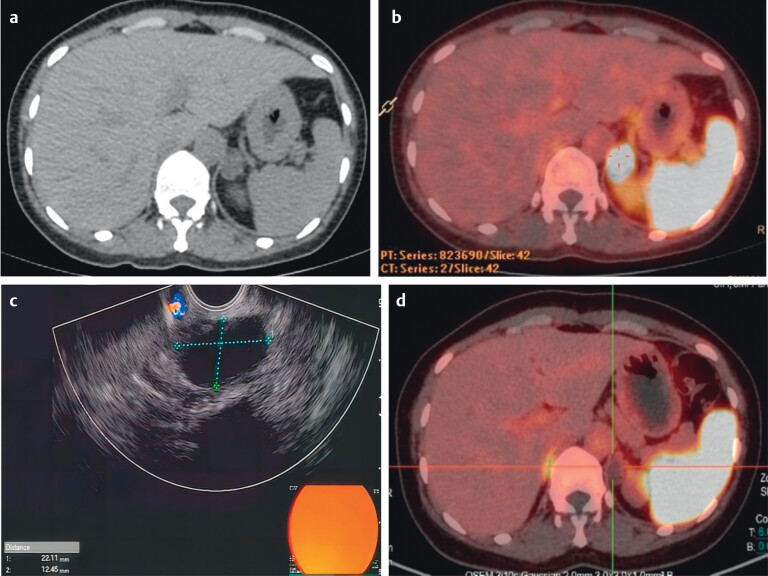
Imaging findings before and after treatment. ( **a** ) CT showing a left adrenal low-density nodule (22×13 mm). ( **b** ) ^68^ Ga-Pentixafor PET/CT demonstrating increased tracer uptake in the lesion before treatment. ( **c** ) EUS showing a hypoechoic lesion in the left adrenal gland (20×19 mm). ( **d** ) Follow-up ^68^ Ga-Pentixafor PET/CT at 6 months showing no significant tracer uptake in the lesion.

The patient declined adrenalectomy; therefore, endoscopic ultrasound (EUS)-guided ethanol ablation was recommended. EUS (Fujifilm 7000 system, Fujifilm, Japan) identified a well-defined 20×19 mm nodule in the left adrenal gland with a safe puncture trajectory avoiding adjacent vascular structures ( Fig. 1 ). Core biopsy was obtained using a 22-gauge needle (Micro-Tech, Nanjing, China). Then, under real-time EUS guidance, ethanol was injected into the hypoechoic nodule through multiple needle tracts. Until a hyperechoic cloud-like change covered the entire nodule, the injection was stopped. A total of 8 mL of ethanol was injected ( Video 1 ). No immediate procedure-related complications occurred. Later, immunohistochemical staining together with Sanger sequencing identified a KCNJ5 mutation ( **Fig.** 2 ), supporting the diagnosis of aldosterone-producing adenoma (APA).

**Video 1** Endoscopic ultrasound-guided ethanol ablation of a left adrenal adenoma for the treatment of primary aldosteronism.


**Fig. 2:**
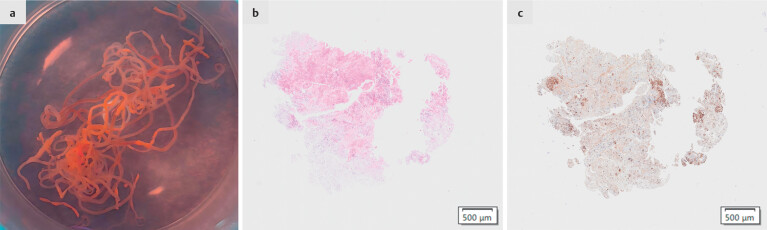
Histopathological and immunohistochemical findings. ( **a** ) An adrenal core biopsy specimen. ( **b** ) Hematoxylin and eosin (H&E) staining. ( **c** ) Immunohistochemical staining for CYP11B2.

The patient&apos;s condition improved obviously on the 1 ^st^ day after the ablation. Without medication, both the blood pressure and serum potassium levels were normal. During the 1, 3 and 6 months of follow-up after the ablation, the patient showed no discomfort and continued to demonstrate a complete clinical and biochemical remission ( **Fig.** 3 ). ​2​ Correspondingly, ^68^ Ga-Pentixafor PET/CT demonstrated no tracer uptake in the lesion.

**Fig. 3:**
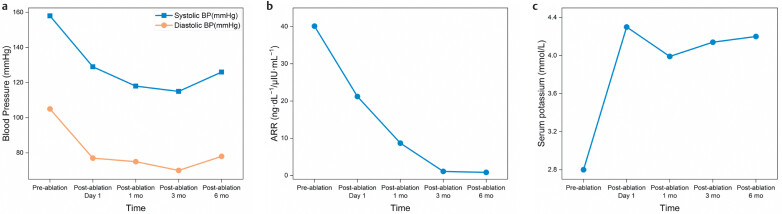
Clinical and biochemical outcomes. ( **a** ) Changes in blood pressure. ( **b** ) Changes in ARR levels. ( **c** ) Changes in serum potassium levels.

Although EUS-guided radiofrequency ablation has been reported for APA, its application is limited by the availability of the ablation needle compatible with EUS in many countries.The present case is the first in which EUS-guided ethanol ablation was used to successfully cure an APA. EUS-guided ethanol ablation might be a safe and feasible minimally invasive alternative in patients with APA who are not suitable for or decline surgery.

Endoscopy_UCTN_Code_TTT_1AS_2AG

## Informed Consent

The patient provided written informed consent for the publication of this report and all associated images. The authors will protect the patient’s personal information and privacy in accordance with all applicable laws and regulations.
